# Effect of Sodium Fluoride Mouthwash on the Frictional Resistance of Orthodontic Wires

**Published:** 2017-09

**Authors:** Allahyar Geramy, Tabassom Hooshmand, Tahura Etezadi

**Affiliations:** 1 Professor, Dental Research Center, Dentistry Research Institute, Tehran University of Medical Sciences, Tehran, Iran; Department of Orthodontics, School of Dentistry, Tehran University of Medical Sciences, Tehran, Iran; 2 Associate Professor, Department of Dental Materials, School of Dentistry, Tehran University of Medical Sciences, Tehran, Iran; 3 Assistant Professor, Department of Orthodontics, School of Dentistry, Mazandaran University of Medical Sciences, Sari, Iran

**Keywords:** Fluoride, Friction, Orthodontics, Saliva

## Abstract

**Objectives:**

The friction between the brackets and orthodontic wire during sliding mechanics inflicts difficulties such as decreasing the applied force and tooth movement and also the loss of anchorage. Therefore, many studies have focused on the factors that affect the friction. The purpose of this study was to assess the effect of 0.05% sodium fluoride mouthwash on the friction between orthodontic brackets and wire.

**Materials and Methods:**

Four types of orthodontic wires including rectangular standard stainless steel (SS), titanium molybdenum alloy (TMA), nickel-titanium (NiTi) and copper-nickel-titanium (Cu-NiTi) were selected. In each group, half of the samples were immersed in 0.05% sodium fluoride mouthwash and the others were immersed in artificial saliva for 10 hours. An elastomeric ligature was used for ligating the wires to brackets. The frictional test was performed in a universal testing machine at the speed of 10 mm/minute. Two-way ANOVA was used for statistical analysis of the friction rate.

**Results:**

The friction rate was significantly higher after immersion in 0.05% sodium fluoride mouthwash in comparison with artificial saliva (P=0.00). Cu-NiTi wire showed the highest friction value followed by TMA, NiTi and SS wires.

**Conclusions:**

According to the results of the current study, 0.05% sodium fluoride mouthwash increased the frictional characteristics of all the evaluated orthodontic wires.

## INTRODUCTION

Sliding mechanics has gained increasing popularity among orthodontists. In this procedure, the wire slides through the brackets and tubes. The sliding resistance between the bracket slot and wire can influence tooth movement; therefore, control of friction during sliding mechanics is one of the most important factors for favorable treatment results [1–3]. The frictional force adversely affects the treatment process through decreasing or even inhibiting tooth movement, which can result in loss of anchorage. According to previous studies, the sliding friction reduces the applied force up to 50% [4–8]. Total elimination of this frictional force is ideal, although it is not practical. Knowledge about the influential factors allows the control of the sliding resistance [4,7,9, 10]. Many parameters influence the estimation of friction rates such as the oral environmental factors, wire and bracket size, wire and bracket material, surface characteristics, type of ligation, and direction and magnitude of force [4,5, 8,9,11]. It is known that the oral environment is not an inert setting and exposes the orthodontic appliances to different materials. Fluoride-containing products are one of the most applicable products during orthodontic treatments.

Since most orthodontic patients are adolescents who usually do not maintain a satisfactory oral hygiene, many clinicians recommend fluoride-containing products to patients [12–15]. The fluoride ion exerts a bactericidal effect by producing hydrofluoric acid (HF). Despite the suitable caries prevention effect of fluoride, production of HF can adversely affect orthodontic wires and brackets [10,13,16]. Previous investigations have shown that fluoride-containing products are destructive, especially for titanium-based alloys. The HF destroys the protective oxide layer on the appliance surface and causes corrosion [6,10,12,16]. The ions released due to corrosion can jeopardize the biocompatibility. Corrosion also alters the surface characteristics, which may lead to changes in the physical properties of the material with a subsequent influence on the clinical efficiency [6,14,17]. In a study by Kao et al [10], the orthodontic wires showed higher friction rates in the acidulated phosphate fluoride (APF) solution. To date, several studies have evaluated the effect of fluoride on the corrosion rate of orthodontic appliances, while investigation on the frictional resistance of wires after the use of fluoridated products is limited, and no study has evaluated copper-nickel-titanium (Cu-NiTi) wires, which are used commonly in low-friction orthodontic treatment systems. The purpose of the present study was to assess the effect of 0.05% sodium fluoride mouthwash on the frictional resistance of different orthodontic wires.

## MATERIALS AND METHODS

In the present study, 80 pieces of orthodontic wires of four different types were studied. The wires included stainless-steel (SS) alloy (Dentsply GAC International, NY, USA), titanium-molybdenum alloy (TMA) (Dentsply GAC International, NY, USA), super-elastic nickel-titanium (NiTi) alloy (Sentalloy, Dentsply GAC International, NY, USA) and copper-nickel-titanium (Cu-NiTi) alloy (Damon optimal-force Copper-NiTi, Ormco, Glendora, CA, USA).

**Fig. 1:**
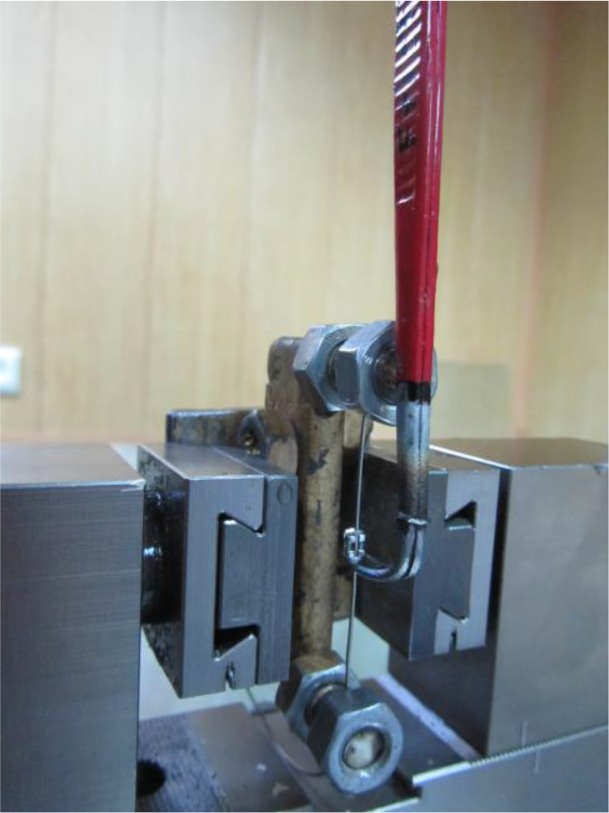
The cling-shaped device used for holding the bracket during the upward movement of the superior jig of the testing machine

The wires were rectangular and with the same dimensions of 0.018×0.025 inch^2^. Twenty pieces of wire with the length of 5cm were used in each of the four groups. Eighty maxillary premolar stainless-steel standard edgewise brackets (American Orthodontics, Sheboygan, Wl, USA) with the slot size of 0.022 inch and without any torque or angulation were used for this study. All the wires and bracket slots were cleaned with alcohol wipes to remove manufacturing dirt and grease. Next, each group of wires was divided into 2 groups, as 10 pieces of each type of wire were placed in the test group and the other 10 pieces were employed in the control group. 0.05% sodium fluoride mouthwash with the pH of 6 (Oral-B, Procter and Gamble Strasse, Gross-Gerau, Germany) was used for evaluating the effect of fluoride. Artificial saliva with the pH of 6.5 was used as control with the following formulation: 2.2mM calcium chloride (CaCl_2_), 2.2mM monosodium phosphate (NaH_2_PO_4_), 50mM acetic acid, 100mM sodium chloride (NaCl), 1 part per million (ppm) sodium fluoride (NaF), and 0.02% sodium azide (NaN_3_). The samples in each group were either immersed in the control solution or in the test solution for 10 hours (10 wires were placed in artificial saliva, while the other 10 wires were immersed in fluoride-containing mouthwash). Afterwards, the samples were placed in an incubator at 37°C. After 10 hours, the samples were immersed in distilled water for 30 minutes. In order to exclude the influence of different elastomeric forces on the results, an elastomeric ligature (American Orthodontics, Sheboygan, Wl, USA) was used to engage the dried wires with brackets by using a special gun type elastomeric ring placer (Hangzhou Sino Ortho Technology Co., Ltd., China). The frictional test was performed in a universal testing machine (Zwick/Roell Z050, Germany). A special fixture and a rigid cling-shaped device (to grab the bracket during the frictional test) were designed and fabricated. The fixture was attached to the inferior jig of the testing machine to fix the wire. Next, the bracket was moved upwards along the 5-mm stretch of wire by the cling-shaped device attached to the superior jig of the machine (Fig. 1) at the speed of 10 mm/minute [10]. Care was taken to align the wire with a completely straight direction during bracket-sliding. The force required to initiate and maintain the bracket movement over the 5-mm test distance was measured. The program was set to highlight the maximum frictional force. Two-way ANOVA was used to compare the friction rate related to the tested solutions and also between the four types of wires. Bonferroni correction was applied as a post-hoc test. The level of significance was set at P<0.05.

## RESULTS

Table 1 shows the frictional force values of the groups categorized by the two different environments of sodium fluoride mouthwash and artificial saliva. According to two-way ANOVA, the frictional force of all the samples significantly increased after immersion in sodium fluoride solution (P=0.00). Different friction values were obtained in the four groups of wires. In both environments (saliva and fluoride solution), Cu-NiTi wire exhibited the highest friction values followed by TMA, NiTi and SS wires. The differences between the frictional forces of different wire types were analyzed by Bonferroni’s test. As shown in Table 2, there were statistically significant differences between SS group and Cu-NiTi and TMA groups (P=0.00), and also between Cu-NiTi and NiTi wires (P=0.00).

**Table 1.** Mean and standard deviation (SD) of the frictional force (N) related to four types of wires in two different environments

	**Wire type**			

	**SS**	**TMA**	**NiTi**	**Cu-NiTi**
0.05% Sodium fluoride mouthwash	1.36±0.18	1.63±0.15	1.45±0.04	1.77±0.23
Artificial saliva	1.14±0.24	1.47±0.14	1.36±0.31	1.64±0.26

SS=Stainless Steel, TMA=Titanium Molybdenum Alloy, NiTi=Nickel-Titanium, Cu-NiTi=Copper-Nickel-Titanium

**Table 2.** Comparison of the frictional force (N) between the four groups of wires

**Wire type**		**Mean Difference**	**Std. Error**	**Sig.**
**SS**	TMA	−0.29^*^	0.07	0<001^*^
	NiTi	−0.15	0.07	0.15
	Cu-NiTi	−0.45^*^	0.07	0<001^*^

**TMA**	SS	0.29^*^	0.07	0<001^*^
	NiTi	0.14	0.07	0.21
	Cu-NiTi	−0.16	0.07	0.12

**NiTi**	SS	0.15	0.07	0.15
	TMA	−0.14	0.07	0.21
	Cu-NiTi	−.30^*^	0.07	0<001^*^

**Cu-NiTi**	SS	0.45^*^	0.07	0<001^*^
	TMA	0.16	0.07	0.12
	NiTi	0.30^*^	0.07	0<001^*^

* Significant

SS=Stainless Steel, TMA=Titanium Molybdenum Alloy, NiTi=Nickel-Titanium, Cu-NiTi=Copper-Nickel-Titanium

## DISCUSSION

The sliding mechanics is the most common mechanics among orthodontists. Friction is one of the most important influential factors in this mechanics because of its adverse effects on sliding. On the other hand, a suitable oral hygiene has always been a big concern for both patients and clinicians. Fluoride-containing products are usually used during orthodontic treatment for protection against caries. Since daily sodium fluoride mouthwash is the most common product suggested by orthodontists, we used this product in our study. Also, we preferred to use an elastomeric ring placement tool for engaging the wire into the brackets instead of the manual approach to ensure similarity between all samples. The present study showed that the friction rate increased in all types of wires after immersion in sodium fluoride mouthwash. This result confirms the result of the study by Kao et al [10]. They revealed that NiTi, TMA and SS wires show higher frictional resistance in the APF solution. In another similar study by Nanjundan and Vimala [18], fluoride mouthwash raised the frictional resistance of SS wires, which is consistent with our results. However, Alavi and Farahi [19] revealed different results. They reported that the friction rates of SS and NiTi wires were higher in the fluoride gel with a high fluoride concentration, but no difference was observed between the fluoride mouthwash with a low fluoride content and the control group. The duration of immersion in the mentioned study was shorter than that in our study (2 minutes per day for two weeks), which makes it difficult to compare the results of the two studies. Abbassy [20] demonstrated that fluoride application does not significantly change the frictional resistance of SS and NiTi wires. This study also used shorter immersion time than ours (1 hour). To date, no other research has evaluated the effect of fluoride on Cu-NiTi wires. In the present experiment, daily sodium fluoride mouthwash increased the frictional resistance of this type of wire, which is commonly used in the self-ligating mechanics. This might have an adverse effect on the self-ligating mechanics, which is known as a low-friction system.

Comparison of the friction rate between four types of wires revealed that Cu-NiTi showed the highest frictional resistance in saliva and sodium fluoride solution, followed by TMA, NiTi and SS wires. This result is in agreement with the findings of previous studies. In a research by Kapila et al [7], round and rectangular TMA wires showed the highest rate of frictional resistance followed by NiTi, whereas SS wire exhibited the lowest amount of frictional resistance. Kao et al [10] also reported that the friction values decreased in TMA, NiTi and SS wires, respectively. Similarly, Fidalgo et al [2] showed that TMA wires have higher frictional resistance than SS wires, irrespective of the bracket system. Further evaluation of Cu-NiTi wires is recommended. The present study has some limitations that should be taken into consideration when interpreting the data. The duration of fluoride application may influence its effect on the wire characteristics. Also, it is difficult to exactly simulate the oral environment. Overall, this study showed that fluoride can raise the friction level during the sliding mechanics, which is worth considering, as it may negatively affect tooth movement, anchorage and treatment results. Further in-vivo investigations are needed to evaluate the precise effect of fluoride on orthodontic wires.

## CONCLUSION

Within the limitations of this study, we concluded that 0.05% sodium fluoride mouthwash significantly increases the frictional resistance between the brackets and SS, NiTi, Cu-NiTi and TMA orthodontic wires, which is worth considering especially in case of critical anchorage. In both environments of sodium fluoride mouthwash and artificial saliva, Cu-NiTi wire exhibited the highest rate of frictional resistance followed by TMA, NiTi and SS wires.
